# Mirror-symmetry protected non-TRIM surface state in the weak topological insulator Bi_2_TeI

**DOI:** 10.1038/srep20734

**Published:** 2016-02-11

**Authors:** I. P. Rusinov, T. V. Menshchikova, A. Isaeva, S. V. Eremeev, Yu. M. Koroteev, M. G. Vergniory, P. M. Echenique, E. V. Chulkov

**Affiliations:** 1 Tomsk State University, pr. Lenina, 36, Tomsk, 634050 Russia; 2 St. Petersburg State University, Universitetskaya nab., 7/9, St. Petersburg, 199034 Russia; 3 Technische Universität Dresden, Bergstraße, 66, Dresden, D-01069, Germany; 4 Institute of Strength Physics and Materials Science, pr. Akademicheskiy, 2/4, Tomsk, 634021 Russia; 5 Donostia International Physics Center (DIPC), Paseo de Manuel Lardizabal, 4, 20018 San Sebastián/Donostia, Basque Country, Spain; 6 Departamento de Física de Materiales, Facultad de Ciencias Químicas, UPV/EHU, 20080 San Sebastián/Donostia, Basque Country, Spain; 7 Centro de Física de Materiales CFM–MPC, Centro Mixto CSIC–UPV/EHU, 20080 San Sebastián/Donostia, Basque Country, Spain

## Abstract

Strong topological insulators (TIs) support topological surfaces states on any crystal surface. In contrast, a weak, time-reversal-symmetry-driven TI with at least one non-zero *v*_1_, *v*_2_, *v*_3_ ℤ_2_ index should host spin-locked topological surface states on the surfaces that are not parallel to the crystal plane with Miller indices (*v*_1_ *v*_2_ *v*_3_). On the other hand, mirror symmetry can protect an even number of topological states on the surfaces that are perpendicular to a mirror plane. Various symmetries in a bulk material with a band inversion can independently preordain distinct crystal planes for realization of topological states. Here we demonstrate the first instance of coexistence of both phenomena in the weak 3D TI Bi_2_TeI which (*v*_1_ *v*_2_ *v*_3_) surface hosts a gapless spin-split surface state protected by the crystal mirror-symmetry. The observed topological state has an even number of crossing points in the 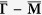 directions of the 2D Brillouin zone due to a non-TRIM bulk-band inversion. Our findings shed light on hitherto uncharted features of the electronic structure of weak topological insulators and open up new vistas for applications of these materials in spintronics.

The gapless spin-polarized states of topological insulators (TIs) at the edge (in two-dimensional (2D) TIs) or at the surface (in three-dimensional (3D) TIs) open up exciting possibilities for applications of these materials in spintronics^1^,^2^,^3^. The 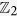 classification of TIs is based on topological invariants (*v*_0_; *v*_1_ *v*_2_ *v*_3_) and allows to attribute 3D TIs to one of two classes. The *v*_0_ = 1 identifies strong TIs, which are characterized by metallic surface states forming an odd number of Dirac cones. These states are robust against perturbations that do not break the time-reversal symmetry. The non-zero *v*_1_, *v*_2_ and *v*_3_ indices define (at *v*_0_ = 0) so-called weak TIs that have an even number of Dirac cones. Spin-locked topological surface states (TSSs) in weak TIs should exist on any crystal surface which is not parallel to the plane with Miller indices (*v*_1_ *v*_2_ *v*_3_)^4^. The surface states of a weak TI exhibit weaker topological protection than those of a strong TI and can be gapped without breaking the time-reversal symmetry^5^.

In contrast to the extensive studies on strong 3D TIs, only a limited number of accounts on electronic properties of weak TIs is available at present, e.g. honeycomb compounds *XYZ* (*X* = K, Na, Li; *Y* = Hg, Cd/Au, Ag; *Z* = Sb, As, P/Te, Se)^6^, a theoretically modelled octahedron-decorated cubic lattice^7^, PbTe/SnTe superlattices^8^, Bi_14_Rh_3_I_9_ 9,10,11, and Bi_2_TeI^12^. With an exception of refs 9,11, the above-mentioned papers do not address the (*v*_1_ *v*_2_ *v*_3_) crystal surface.

Classes of materials with non-trivial band structures are not restricted to 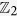 TIs. So-called topological crystalline insulators (TCI) represent another type of topological insulating materials with a band gap inverted by the strong spin-orbit coupling (SOC). However, the topological phase therein is protected by a symmetry differing from the time-reversal symmetry, namely by the crystal mirror symmetry^13^,^14^. Consequently, these materials possess topologically protected surface states on the surfaces that are perpendicular to the mirror plane.

It was established that the majority of known strong 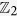 TIs, like Bi_2_Te_3_ with the trigonal crystal structure, also accommodate a TCI phase and, thus, the Dirac state residing on the (111) surface is simultaneously protected by both the time-reversal and the crystal mirror symmetries. A magnetic field applied perpendicular to the mirror plane in Bi_2_Te_3_ destroys the 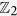 topological phase and, hence, unravels the dual topological nature of the 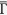 Dirac state since the TCI phase is preserved and the Dirac surface state remains gapless^15^.

From this viewpoint, weak TIs are perspective for realization of a dual topological phase where the Dirac surface state of the 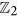 topological phase and the topological states protected by the crystal symmetry can be manipulated separately without applying a magnetic field, owing to the fact that they appear at different crystal surfaces. In the following, we show that the weak 3D TI Bi_2_TeI is a promising candidate.

This chemically stable crystalline compound is built by a periodic stack of two types of 2D fragments: BiTeI trilayers and Bi-bilayer^16^. Previously it was theoretically predicted that Bi_2_TeI is a weak TI characterized by the (0; 0, 0, 1) 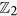 invariant^12^. Accordingly, calculations of the electronic structure of the (010) surface, which is not a natural cleavage surface of the compound, revealed TSS inside the bulk gap^12^.

Herewith we present an *ab-initio* density-functional-theory study of the electronic structure of the Bi_2_TeI natural cleavage (001) surface with different types of possible terminations. By means of these simulations we demonstrate for the first time that the (*v*_1_ *v*_2_ *v*_3_) surface of a weak 3D TI can accommodate a gapless spin-split surface state in the band gap. The preferable Te termination holds topological surface state, which opposite-spin branches cross at non-symmetric points of the 2D Brilloin zone (BZ) lying in the 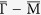 directions (≈0.3 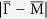) where they are protected by mirror symmetry of the system of the system. Away from the 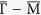 mirror plane a tiny gap of ≈8 meV opens up since the crossing is avoided by a symmetry constraint. On the contrary, the iodine- and [Bi_2_]-terminated surfaces of Bi_2_TeI exhibit Rashba-like spin-split bands in the BZ center in addition to an even number of gapless 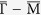 TSSs.

## Results

**Figure 1:**
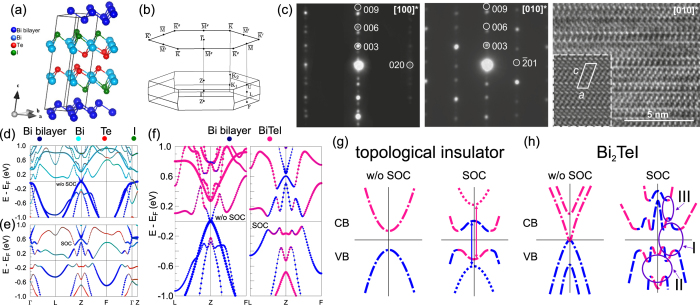
Crystal structure and bulk electronic spectrum of the Bi_2_TeI. (**a**) A view of the Bi_2_TeI crystal structure. (**b**) The 3D and 2D Brillouin zones of a Niggli-reduced cell. (**c**) Experimental diffraction patterns of the [100]* (left) and the [010]* (centre) zones for a 50 nm thick lamella cut out from a Bi_2_TeI crystal. Right: a HRTEM image of the [010]* zone with an inserted simulated HRTEM (*d* = 28 nm, *t* = 10 nm) outlined by a white dashed line. According to simulations, the bright spots correspond to atoms. The unit cell is outlined by a solid white line. Bulk band spectra of Bi_2_TeI calculated without (**d**) and with (**e**) SOC included. The atomic contributions are color-coded. (**f**) The same spectra magnified in the vicinity of the Z point. Deep-pink and blue colors denote the states localized in the BiTeI trilayer and the Bi-bilayer, respectively. A schematic view of the inversion of the bulk-band-gap edges for 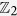 Topological Insulator (**g**) and Bi_2_TeI (**h**). The bands with opposite parities are outlined by dashed and dotted lines; the violet ovals I–III indicate areas of the band inversion.

The crystal structure of Bi_2_TeI was elucidated from a single-crystal X-ray diffraction experiment^16^. The compound crystallizes in a centrosymmetric monoclinic unit cell (space group *C*12/*m*1, a = 7.586 Å, b = 4.380 Å, c = 17.741 Å, *β* = 98.20°) with 16 atoms which can be represented as a Niggli-reduced cell (a = b = 4.380 Å, c = 17.741 Å, *α* = *β* = 82.9047°, *γ* = 60.0025°) with 8 atoms [Fig. 1(a)]. We performed crystal growth experiments based on the synthetic protocol from^16^ and determined that the stacking sequence of layers and the lattice parameters are in agreement with the earlier reported results.

The layered Bi_2_TeI structure comprises two 2D building blocks relevant for TI studies: the Bi-bilayer, [Bi_2_], which is theoretically predicted to be a 2D TI^17^,^18^, and BiTeI trilayer, which is a structural unit of the BiTeI compound with giant Rashba-like spin splitting^19^,^20^,^21^,^22^,^23^,^24^. The BiTeI compound can be tuned into a TI phase by external pressure^25^. Moreover, a single BiTeI trilayer holds the giant Rashba state itself^26^. These two types of fragments alternate in the stack in such a manner that the Bi-bilayers are always inserted between iodine atomic layers forming [Te-Bi-I] · [Bi_2_] · [I-Bi-Te] sandwiches. In contrast to covalent bonding within the [Bi_2_] and the [I-Bi-Te] fragments, the sandwiches are held together by significantly weaker van-der-Waals interactions. As a result, the natural cleavage surface for Bi_2_TeI is perpendicular to the (001) direction, and this ensures absence of trivial surface states within the bulk band gap. The material can be easily cleaved with scotch tape into thin flakes with three possible terminations: tellurium or iodine planes of the [Te-Bi-I] block, or bismuth bilayers. Our experimental results on cleavage show that the Te termination is predominant (more than 80% of instances). Furthermore, we estimated energies for the Te-Te and the Bi_2_-I cleavages and found that the cleavage between adjacent [Te-Bi-I] · [Bi_2_] · [I-Bi-Te] sandwiches, is ≈50 times more favorable than that between the [Bi_2_] and the [I-Bi-Te] fragments, which is consistent with the experimentally found preference for the Te termination.

Stacked layers in the periodic Bi_2_TeI structure are slightly shifted with respect to each other in the *ab* plane (this shift amounts to ca. 0.001 Å only within the unit cell). This forces the reduction of the trigonal point symmetry of the [Bi_2_] and [I-Bi-Te] building blocks, respectively, down to the monoclinic symmetry of the entire Bi_2_TeI crystal lattice. As a result, the BZ of the (001) surface is a slightly distorted hexagon with the base angles equal to 120.005 and 119.995 degrees, respectively. For this reason, the crystal structure can be regarded as a pseudo-hexagonal one in which only one mirror plane, 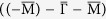, is retained, whereas the other two mirror planes of the hexagonal lattice are transformed into pseudo-mirror planes (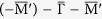 and 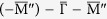), see Fig. 1(b). Below the influence of this small distortion from the trigonal symmetry on the electronic structure is discussed.

Spin-orbit interaction (SOC) plays a crucial role in this material. The electronic spectrum of Bi_2_TeI without SOC included (Fig. 1(d)) has semimetallic character with a zero gap at the Z point. Switching on SOC transforms the electronic spectrum of Bi_2_TeI from a semimetallic to an insulating one (Fig. 1(e)). Our calculation of the 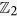, determined from the product of the parity eigenvalues of the occupied states at the time-reversal invariant momenta (TRIM), confirms the (0; 0, 0, 1) indices obtained by Tang *et al.*^12^. However, the SOC-induced band inversion is more complicated in this case than in conventional TIs, like Bi_2_Te_3_. In the latter, SOC lifts up the Kramers degeneracy in a valence-band-edge state (formed by Te *p*-orbitals) and a conduction-band-edge state (formed by Bi *p*-orbitals) at the TRIM. Since the strength of SOC is larger than the gap width, it leads to an inversion of the edge bands and formation of a new insulating gap owing to their hybridization (see a schematic picture in Fig. 1(g)). As can be seen from Fig. 1(d,e), the SOC-induced band inversion for the Bi_2_TeI cannot be easily described in terms of atomic orbitals. Nevertheless, if one combines the orbitals that belong to different blocks (Fig. 1(f)) it can be deduced, firstly, that the bulk-band inversion in Bi_2_TeI occurs between the states of the [Bi_2_] and the [BiTeI] structural blocks, and, secondly, that two pairs of bands contribute to the SOC-induced band inversion in the resulting spectrum (shown schematically in Fig. 1(h)), in contrast to conventional TIs. The consequence of such complicated band inversion is that the band-gap-inversion area is not centered at the TRIM (see the indicated area I in Fig. 1(h)), as in the case of conventional TI. Furthermore, it triggers formation of additional hybridization gaps between the inverted bands in the valence and conduction bands (areas II and III, respectively). Based on these premises, emergence of a band-gap topological surface state at non-symmetric points as well as the valence- and conduction-band TSSs can be expected.

**Figure 2:**
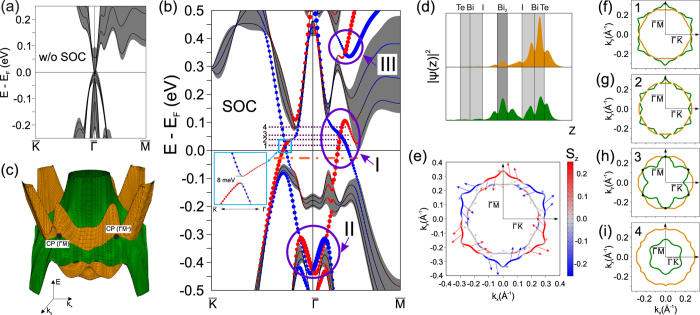
Electronic structure of the Te-terminated surface. The band structure of a Te-terminated Bi_2_TeI (001) slab (black lines) and the projected bulk band structure (gray background) without (**a**) and with (**b**) spin-orbit coupling. The dots in the panel (**b**) represent weights of the states in the outer layers of the slab multiplied by a value of in-plane spin components *S*_*x*_ and *S*_*y*_ (red and blue colors denote the positive and negative projections of the spin vector 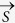 on Cartesian axes, respectively). The violet ovals indicate: the gapless band-gap (I), the valence-band (II), and the conduction-band (III) TSSs. The inset shows a magnified view of the 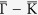 tiny gap in the band-gap TSS. (**c**) A 3D view of the band-gap topological surface state. (**d**) Spatial distribution of the charge density integrated over (x, y) planes, 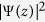, for both branches of the band-gap TSS. Yellow and green colors accord with those in the panel (**c**). (**e**) Spin-resolved constant-energy contours (CECs) taken in the middle of the bulk gap (see the orange dash-doted line in (**b**)). (**f**–**i**) CECs for the cuts 1–4 defined in the panel (**b**).

Simulations of the preferable Te-terminated surface were performed on a slab composed of four [Te-Bi-I] · [Bi_2_] · [I-Bi-Te] sandwiches, i. e. of 32 atomic layers. Figure 2(a) shows the surface band-structure calculated without inclusion of SOC. This spectrum exhibits semimetallic character, so that the valence and conduction bands touch only at the 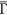 point and no surface states exist, as can be expected for a surface formed by cleavage through a van-der-Waals gap. The surface spectrum with included SOC (Fig. 2(b)) features a spin-polarized TSS that crosses the band gap along the 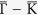 and 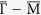 directions. Besides, additional spin-split TSSs arise in the valence and conduction bands. They are marked by violet circles II and III, respectively, in Fig. 2(b). Henceforward we focus solely on the band-gap TSS. This state is gapless with a crossing point (CP) lying at ≈0.3 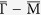 near the conduction band, whereas in the 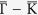 direction it has a tiny gap of 8 meV (Fig. 2(b)). Noteworthy is that the TSS comprises two degenerate states, one for each slab surface. For the considered slab thickness these states are slightly coupled, introducing an artificial minigap (of 2 meV) in the crossing point along the 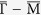 direction. The doubling of the slab in the *z* direction halves the size of this minigap, whereas the gap in TSS along the 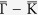 remains unchanged. Moreover, identical minigaps are also found in the 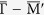 and 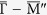 directions, thus leading to a conclusion that slight deviations from the trigonal structure in the title compound affect the energy spectrum even weaker than artifacts of the slab model. From these finding we can infer that the crystal-symmetry protection is tolerant (at least for the slab geometry) toward small structural distortions.

Energy dependence of the TSS in the full 2D BZ shows that the spin branches with opposite spins form two continuous surfaces with different spatial localization around the center of the BZ (see Fig. 2(c)). One surface (highlighted in yellow) is strongly localized in the outer [Te-Bi-I] trilayer, while the other one (in green) is localized in both [Te-Bi-I] and adjacent [Bi_2_] blocks (see Fig. 2(d)). There is a tiny gap at the intersection of these two branches at all 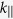 away of the 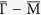 mirror-planes, where the crossing is avoided by a symmetry constraint^27^. The TSS remains gapless only in six CPs lying along the 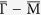 directions (marked by black dots in Fig. 2(c)), where it is protected by the crystal mirror symmetry.

The 2D Fermi Surface (FS) is composed of two Γ-centered contours (Fig. 2(e)) for various positions of the chemical potential within the entire bulk band gap. While the inner contour is almost circular, the outer one is subject to strong hexagonal warping. The inner branch of the TSS exhibits clockwise spin-rotation with a negligible out-of-plane spin component, whereas the outer, hexagonally warped branch demonstrates generally counter-clockwise helicity with a more complex spin-texture.

Let us consider the evolution of the 2D FS topology of the TSS at the energies above the conduction band minimum. Right above *E* = 0, where the TSS has a tiny gap in the 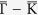 directions (see cut 1 in Fig. 2(b,f)), two 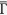-centered contours transform into six pockets. At higher energies (cut 2 in Fig. 2(b,g)), the tiny TSS gap shifts away from the point on the high-symmetry direction which initiates further transformations in the FS from six to twelve pockets. Each pocket is centered at the points residing in high-symmetry directions. At *E* = *E*_*CP*_ (cut 3 in Fig. 2(b,h)) the FS again evolves into two distinct 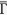-centered contours so that the inner, camomile-like contour touches the outer one at the points lying along the 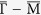 directions. Further shift of the chemical potential towards higher energies (cut 4 in Fig. 2(b,i)) leads to contraction(expansion) of the inner(outer) FS contour.

**Figure 3:**
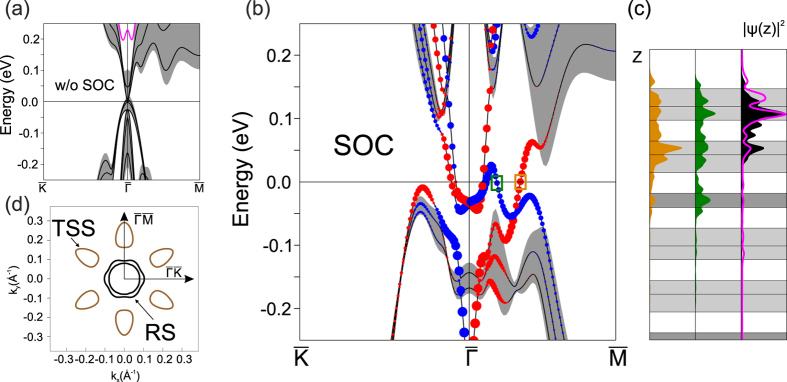
Electronic structure of the iodine-terminated surface. An energy spectrum of the iodine-terminated surface with spin-orbit coupling switched off (**a**) and on (**b**). (**c**) Spatial distribution of the charge density 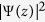 for the TSS branches corresponding to the positive (orange) and negative (green) spin projections (note the small colored squares in (**b**)) and for the trivial surface state at 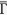 with (black shading) and without (deeppink curve) including SOC. (**d**) Constant energy contours at the center of the bulk band gap.

Let us now regard the less frequent iodine-terminated surface of Bi_2_TeI. This case was approximated as an [I-Bi-Te] overlayer on top of the Te-terminated slab, so we consider mainly the changes that this add-on introduces in the electronic structure of the Te-terminated surface. The surface spectrum calculated without taking spin-orbit coupling into account (see Fig. 3(a)) is generally similar to the Te-terminated surface spectrum (see Fig. 2(a)) with an exception of a surface state residing at the 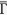 point at ≈0.2 eV in a local gap of the conduction band (marked by a deep pink curve). This state originates from the splitting of the upper edge of the first conduction band which is caused by positive band bending that is provided by the [I-Bi-Te] overlayer. This case bears similarity to the effects introduced by the iodine-terminated surface in the BiTeI compound^21^.

The switched-on SOC provokes significant modification of the surface electronic structure as compared to the case of the Te-terminated surface. First of all, SOC induces the bulk-band inversion, so that the bulk conduction band, from which the surface state is split off, dives into the valence band and, consequently, a trivial surface state emerges in the band gap (Fig. 3(b)). In spite of this modification the trivial surface state maintains its localization within the surface trilayers with a maximum in the outer trilayer (Fig. 3(c), deep pink and black curves in the outmost right panel) regardless of whether SOC is taken into account or not. Another consequence of the activated SOC is emergence of a Rashba-type spin splitting at small 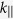 for the trivial surface state at 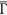. At larger 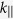 the splitting acquires a more complicated character owing to hybridization of the trivial surface state with the topological one. This hybridization also substantially modifies the gapless spin-helical topological surface state which survives at 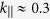 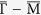, although the energy of the CP noticeably lowers (with respect to its position at the Te-terminated surface) and approaches the bulk valence band (Fig. 3(b)). As far as localization of the TSS is concerned, the spatial distribution profiles for the opposite spin branches (Fig. 3(c), yellow and green curves) resemble those at the Te-terminated surface (Fig. 2(e)) with the difference that now they penetrate considerably into the [I-Bi-Te] overlayer.

Strong alternation of the electronic spectrum of the Bi_2_TeI surface induced by the [I-Bi-Te] overlayer causes substantial changes in the 2D Fermi surface. In contrast to two 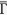-centered contours provided by the topological surface state (Fig. 2(f)), it is now formed by six isolated egg-shaped pockets enclosing the TSS degeneracies plus two concentric contours from the Rashba-like trivial surface state (RS) in the vicinity of 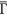 (see Fig. 3(d)).

The last possible cleavage surface of Bi_2_TeI is terminated by the Bi-bilayer and can be approximated as a [Bi_2_] · [I-Bi-Te] overlayer on top of the Te-terminated surface or, alternatively, as a [Bi_2_] overlayer on top of the iodine-terminated surface.

**Figure 4:**
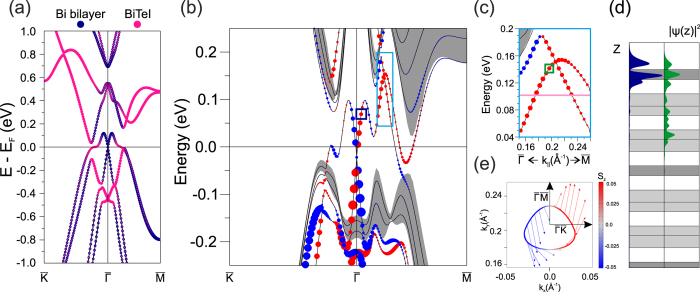
Electronic structure of a free-standing [Bi_2_] · [I-Bi-Te] film and the Bi_2_-terminated surface. (**a**) Electronic spectrum of a [Bi_2_] · [I-Bi-Te] film. (**b**) Band structure of the Bi_2_-terminated surface. (**c**) A magnified view of topological surface state in the 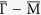 direction (note a light blue rectangle in the panel (**b**)). (**d**) Charge density distribution of the Rashba-like and topological surface states. The color scheme accords with the colors of the small squares in the panels (**b**,**c**). (**e**) Spin-resolved energy contour for 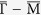 topological surface state below the CP (note a pink line in the panel (**c**)).

First, the electronic structure of a freestanding [Bi_2_] · [I-Bi-Te] overlayer is addressed. As can be seen from Fig. 4(a), its spectrum has a gap at the 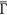 point with a valence-band Rashba-split state at the Fermi level that is mostly localized in the [Bi_2_]-block in the vicinity of the 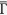 point, while away from this point the [I-Bi-Te] trilayer contributes predominantly to this state.

The Rashba-like state formed by the [Bi_2_] · [I-Bi-Te] overlayer at the Fermi level is retrieved as the most prominent feature in the surface spectrum of the Bi_2_-terminated Bi_2_TeI. Comparison of the spectra of a free-standing [Bi_2_] · [I-Bi-Te] overlayer and the Bi_2_-terminated surface shows that the dispersion and spatial localization of the discussed state do not change (Fig. 4(d), left) as it remains localized in the [Bi_2_] block. The major difference is, however, that the spectrum is gapless in the case of the Bi_2_-terminated surface (see a light blue rectangle in Fig. 4(b)). This state with the CP at ≈0.25 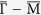 (Fig. 4(c)) below the conduction band can be regarded as the survived topological state which penetrated deeply into the adjacent sublayers down to a second trilayer (Fig. 4(d), right). The spin texture of this state is highly unusual because of the strong hybridization with the trivial Rashba state (Fig. 4(e)), namely, the spin, being mostly in-plane, always has a positive *S*_*x*_ component around the contour. The counterpart gapless state located in the 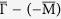 direction has an opposite spin direction and, thus, the net spin equals zero over the Brillouin zone.

## Methods

### Crystal growth

A stoichiometric mixture of Bi, Te and BiI_3_ (sublimated in vacuum prior to use) was placed into a silica ampoule that was then evacuated and sealed. The ampoule was heated to 823 K with a rate of 10 K/h, tempered for 2 hours and subsequently cooled down to ambient temperature with a rate 2 K/h. Largely overgrown crystalline platelets were found on the batch along with some single crystals that grew on the ampoule’s walls. The products were characterized by semi-quantitative energy dispersive X-ray analysis (SU8020 (Hitachi) SEM, Silicon Drift Detector (SDD) *X* − *Max*^*N*^ (Oxford)), X-ray diffractometry (X’Pert Pro MPD diffractometer (PANalytical), Ge(111) monochromator, Cu-*K*_*α*_ radiation) and TEM methods (FEI Titan F20 microscope with CS-correction operating at 80 kV). A typical SAED and HRTEM images for Bi_2_TeI are given in Fig. 1(c) of the main text. The observed stacking sequence of layers and lattice parameters are in consistent with the those determined from the structure elucidation in ref. 16.

### DFT calculations

Electronic structure calculations were carried out within the density functional theory using the projector augmented-wave method^28^ as implemented in the VASP code^29^,^30^ and ABINIT code^31^. The PAW data sets in ABINIT code were taken from ref. 32. The exchange-correlation energy was treated using the generalized gradient approximation^33^. The Hamiltonian contained the scalar relativistic corrections and the spin-orbit coupling was taken into account. The calculation of 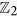 invariant was carried by using the parity of the wave functions obtained in the framework of the Full Potential Linearized Augmented Plane wave (FLAPW) method implemented in FLEUR code^34^. The bulk and surface spectra obtained using different codes are in full agreement.

## Additional Information

**How to cite this article**: Rusinov, I. P. *et al.* Mirror-symmetry protected non-TRIM surface state in the weak topological insulator Bi_2_TeI. *Sci. Rep.* **6**, 20734; doi: 10.1038/srep20734 (2016).
